# The virulence phenotypes and molecular epidemiological characteristics of *Vibrio fluvialis* in China

**DOI:** 10.1186/1757-4749-5-6

**Published:** 2013-03-22

**Authors:** Pu Liang, Xiaoying Cui, Xiaoli Du, Biao Kan, Weili Liang

**Affiliations:** 1 State Key Laboratory for Infectious Disease Prevention and Control, and National Institute for Communicable Disease Control and Prevention, Chinese Center for Disease Control and Prevention, Beijing, 102206, People’s Republic of China

**Keywords:** *Vibrio fluvialis*, VFP, Cytotoxicity, Antibiotic Susceptibility, PFGE, Biofilm, Virulence

## Abstract

**Background:**

*Vibrio fluvialis* is considered to be an emerging foodborne pathogen and has been becoming a high human public health hazard all over the world, especially in coastal areas of developing countries and regions with poor sanitation. The distribution of virulence factors, microbiological and molecular epidemiological features of *V. fluvialis* isolates in China remains to be examined.

**Methods and results:**

PCR targeted at the virulence determinants and phenotype tests including metabolism, virulence and antibiotic susceptibility were performed. Pulsed-field gel electrophoresis (PFGE) analysis was used to access the relatedness of isolates. A strain with deletion of the arginine dihydrolase system was first reported and proved in molecular level by PCR. Virulence genes *vfh*, *hupO* and *vfpA* were detected in all strains, the ability to produce hemolysin, cytotxin, protease and biofilm formation varied with strains. High resistance rate to β-lactams, azithromycin and sulfamethoxazole were observed. Twenty-seven percent of test strains showed resistant to two and three antibiotics. PFGE analysis demonstrated great genetic heterogeneity of test *V. fluvialis* strains.

**Conclusion:**

This study evaluated firstly the biological characteristics and molecular epidemiological features of *V. fluvialis* in China. Some uncommon biochemical characteristics were found. Virulence genes were widely distributed in the isolates from patient and seafood sources, and the occurrence of virulence phenotypes varied with strains. Continued and enhanced laboratory based-surveillance is needed in the future together with systematically collection of the epidemiological information of the cases or the outbreaks.

## Background

*V. fluvialis* is considered to be an emerging foodborne pathogen and has been implicated in outbreaks and sporadic cases of acute diarrhea [1]–[7]. Besides, *V. fluvialis* posed a significant economic threat for aquaculture for being pathogenic for cultured fish and lobsters [8]. Gastroenteritis caused by this organism is associated with drinking of contaminated water or consumption of raw or improperly cooked seafood [3,5]. In addition, *V. fluvialis*-associated extraintestinal infections, such as hemorrhagic cellulites and cerebritis [9], peritonitis [10], acute otitis [11], biliary tract infection [12], bacteraemia [13] and even ocular infections [14] were also reported.

Several toxins that may be important in pathogenesis have been reported in *V. fluvialis* including a Chinese hamster ovary (CHO) cell elongation factor, CHO cell-killing factor, enterotoxin-like substance, lipase, protease, cytotoxin, and hemolysin [15]–[20]. The cell-free culture filtrates of *V. fluvialis* strains were capable of evoking distinct cytotoxic and vacuolation effects on HeLa cells [21]. The heme utilization protein gene *hupO* in *V. fluvialis* was induced under iron-restricted conditions and is associated with virulence expression through stimulation of hemolysin production and resistance to oxidative stress [22]. In spite of many pathogenic factors were characterized, their precise role in producing the clinical manifestations remains to be known and little definitive information about the pathogenic mechanism of *V. fluvialis* has been achieved.

The largest outbreak of *V. fluvialis* infection was reported in Bangladesh between October 1976 and November 1977, with more than 500 patients [2]. In the United States, *V. fluvialis* accounted for 10% of vibrio-caused clinical cases along the Gulf Coast [23]. *Vibrio* surveillance data show that it was responsible for 82 of the 1,584 *Vibrio* infections reported to the Centers for Disease Control and Prevention during 1997–2000 [24]. Srinivasan *et al.* reported that *V. fluvialis* is being isolated with an increased frequency from hospitalized patients in Kolkata, India, with cholera-like illnesses [25]. Study of Ramamurthy group revealed the isolation rate of *V. fluvialis* increased from 0.7% in 2002 to 2.2% in 2009 [4]. Recently, it was reported that 3,529 (91.2%) of 3,871 residents of Pakhirala village of the coastal islands of the Sunderbans, situated in the southern part of West Bengal, eastern India, were affected by watery diarrhea caused by *V. fluvialis* within a span of six weeks following Cyclone Aila in 2009 [5]. Furthermore, *V. fluvialis* behaved more aggressively than *V. cholerae* O1 in an epidemic situation with a higher attack rate and a different clinical picture [5]. In general, the clinical symptoms of the gastroenteritis caused by *V. fluvialis* are similar to those caused by *V. cholera*, including mild to moderate dehydration, abdominal pain, vomiting, fever, and diarrhea with presence of blood which is a notable different from cholera [5,26]. The infection of *V. fluvialis* is generally common in infants, children, and young adults [2,21,27]. Therefore, *V. fluvialis* has been becoming a high human public health hazard all over the world, especially in coastal areas of developing countries and regions with poor sanitation.

In China, the etiological characteritics of *V. fluvialis* and its epidemiology of infection were not even fairly known with little information in the literature. 4.8% isolation rate of *V. fluvialis* was reported in sea products [28]. Considering the occurrence and prevalence of *V. fluvialis* enteritis in different developed and developing countries [2,6,7,27,29,30], the infection of *V. fluvialis* in China is probably undetected due to complexity in the identification and less attention in the surveillance. Historically, only toxigenic *V. cholerae* serogroups O1 and O139 were nationally notifiable. *V. fluviali*s phenotypically resembles Aeromonas species [26,28], and is closely similar to *V. furnissii* which is aerogenic in nature [31]. Many questions remain unanswered about its microbiological characteristics, distribution of virulence factors, mechanism of pathogenicity and epidemiology of the infection. The objective of this study was to investigate and compare the virulence determinants, drug resistance profiles of 43 *V. fluvialis* isolated in China. Main biological characteristics and clonal relationship among the strains were examined.

## Methods

### Bacterial strains and culture condition

A total of 43 strains of *V. fluvialis* collected from six different geographical regions in China were included in this study. All strains were maintained in Luria-Bertani (LB) broth supplemented 15% glycerol and stored at −80°C. Of these, 10 were isolated from marine products including shrimp and fish. The rest 33 were isolated from the stool samples of diarrheal patients. Strain CICC21612 was acquired from the National Institutes for food and drug Control (NIFDC) and used as the reference strain of *V. fluvialis.* In general, *V. fluvialis* strains were grown in LB broth containing 1% NaCl with shaking at 37°C. The detailed information of every strain was showed in Additional file 1: Table S1.

### Polymerase chain reaction (PCR)

PCR was used to confirm the identities of presumptive *V. fluvialis* by using two species-specific primers *toxR*-F/*toxR*-R and VFLU-F/VFLU-R which targeted at the *toxR* gene and 16S-23S rDNA intergenic sequence [32,33]. PCR assays were also performed to screen the presence of the virulence genes *vfh*, *hupO*, *vfpA* and *stn*, *int* IV gene specific for the class IV integron [34], *sul*II gene encoding for the sulfamethoxazole resistance [35]. Based on sequence of *V. furnissii* NCTC 11218 [36], primers *arc*-F/*arc*-R were designed to amplify the arginine dihydrolase system in *V. flluvialis*. A water-boiled template of each strain was used in the all PCR assays. Each PCR involved an initial denaturation at 94°C for 4 min, followed by 33 cycles each consisting of an initial denaturation at 94°C for 40 sec followed by annealing and extension steps. Final polymerization was included at 72°C for 6 min. Primers and the corresponding annealing temperatures were listed in Table 1.

**Table 1:** Primers and amplification conditions used in this study

**Primer**	**Sequences (5’-3’)**	**Target size (bp)**	**Annealing temp. (°C)**	**Reference**
*toxR*-F	GACCAGGGCTTTGAGGTGGACGAC	217	65	[32]
*toxR*-R	AGGATACGGCACTTGAGTAAGACTC			
VFLU-F	ATAAAGTGAAGAGATTCGTACC	278	60	[33]
VFLU-R	GTATTCCTGAATGGAATACAC			
*Int* IV-F	AACACCGCTTGCACCTCTAT	525	53	[34]
*Int* IV-R	TGTATGCGCTTGAGAGTCC			
*stn*-F	GGTGCAACATAATAAACAGTCAACAA	375	53	[44]
*stn*-R	TAGTGGTATGCGTTGCCAGC			
*sul*II-F	AGGGGGCAGATGTGATCGAC	606	55	[35]
*sul*II-B	TGTGCGGATGAAGTCAGCTCC			
*vfh*-F	GCGCGTCAGTGGTGGTGAAG	800	61	This study
*vfh*-R	TCGGTCGAACCGCTCTCGCTT			
*hupO*-F	ATTACGCACAACGAGTCGAAC	600	56	This study
*hupO*-R	ATTGAGATGGT AAACAGCGCC			
*vfpA* -F	TACAACGTCAAGTTAAAGGC	1790	55	This study
*vfpA* -R	GTAGGCGCTGTAGCCTTTCA			
*arc-*F	AGTTTATGCGTCTGGCTTG	3427	56	This study
*arc*-R	ATGAGTAAGTTATACGTAGG			
*arc*-rev	GCTTCGGCCCACATAATAA (paired with *arc*-F)	2170	56	This study
*arc*-ck-up	TTACCACCTAATGCGACGA (paired with *arc*-R)	1235	56	This study

### Biochemical characteristics of *V. Fluvialis*

Molecular confirmed *V. fluvialis* strains were plated on LB agar and thiosulphate citrate bile salts sucrose agar (TCBS) followed by incubation at 37°C overnight. API 20E (bioMérieux) identification strip was used to characterize the biochemical features. The string test was performed using 0.5% sodium deoxycholate solution with fresh colonies grown on LB agar. Cytochrome oxidase was detected using Oxidase Reagent (bioMérieux). Susceptibility to 10 μg of vibriostatic compound O/129 (2, 4-diamino-6, 7-diisopropylpteridine phosphate) was determined in LB agar [38]. Salt tolerance was determined by growing the strains in LB broth overnight with shaking at 37°C without NaCl or with 6% or 7% NaCl.

### Haemolysin assay

The ability of *V. fluvialis* to produce extracellular hemolysin (VFH) was examined on Columbia blood agar containing sheep erythrocytes. Fresh single colonies of each strain from LB agar were spotted onto blood agar plates and incubated at 37°C for 24-72 h. The appearance of hemolytic zone was observed per 24 h.

### Haemagglutinin activity

Fresh colonies of each strain from LB agar were suspended in PBS and cell density of the suspension was adjusted to 10^5-6^ cfu/ml. Chicken and human erythrocytes were washed respectively and then diluted to a final concentration of 1.5% (vol/vol) in sterile 10 mM PBS (pH 7.0). 100 μl of the cell suspension was mixed with 100 μl of 1.5% chicken erythrocytes in 8-well 200 μl PCR tubes (Axygen, Germany). The mixture was incubated at 25°C for 45 min, and agglutination was monitored visually. PBS was used as negative control, *V. cholerae* strain N16961 was used as positive control.

### Metalloprotease activity

*V. fuvialis* protease (VFP) activity was measured using an azocasein assay [39]. Briefly, *V. fuvialis* strains were cultured overnight in LB broth at 37°C with agitation. 100 μl of azocasein (5 mg/ml) in 100 mM Tris (pH 8.0) was incubated with 100 μl of cell culture supernatants for 1 h at 37°C. The reaction was stopped by adding 400 μl of 10% trichloroacetic acid solution. After centrifugation for 15 minutes at 13000 rpm, the trichloroacetic acid supernatant was transferred to a new tube containing 700 μl of 525 mM NaOH, and the optical density was determined at 442 nm (OD442). One azocasein unit is the amount of enzyme that produces an increase of 0.01 optical density units per h. Three independent cultures for each strain were tested and LB broth was used as blanks.

### Biofilm formation

Biofilm formation was measured by the crystal violet staining method [40]. Overnight cultures of each strain were diluted 1:50 in fresh medium and 100 μl of dilution transferred to 96-well flat-bottom microtitre plates. The plates were incubated statically for 24 h at 30°C for biofilm development. At the desired end-point, OD600 was determined and the plates were rinsed with PBS buffer for to remove the non-adherent cells. Biofilms were stained with 120 μl 0.1% crystal violet for 30 min at 30°C followed by rinsing four times with PBS. The cell-associated dye was solubilized in 120 μl of dimethyl sulfoxide (DMSO) and quantified by measuring the OD570 of the resulting solution. Final results were normalized for growth and expressed as the A570/OD600 ratio. Each assay was performed in triplicate.

### Antibiotic susceptibility test

Antibiotic susceptibility test was performed using the microbroth dilution method according to the guidelines of the current Clinical and Laboratory Standards Institute (CLSI). All *V. fluvialis* strains were tested for susceptibility to 15 antibiotics which include ampicillin, amoxicillin/clavulanic acid, cefotaxime, ceftriaxone, ceftazidime, chloramphenicol, ciprofloxacin, gentamicin, nalidixic acid, streptomycin, sulfamethoxazole, trimethoprim, co-trimoxazole, tetracycline, and azithromycin. Multidrug resistance was defined as a presence of resistance to two or more classes of antibiotics. *Escherichia coli* ATCC 25922 was used for quality control. No interpretive criteria for *V. fluvialis* were available based on CLSI guidelines, the minimum inhibitory concentrations (MICs) of antibiotics were determined by referring to the CLSI standards for *V. cholerae* if available; otherwise breakpoints for *Enterobacteriaceae* were applied.

### Tissue culture assay

Human laryngeal carcinoma Hep-2 cells were grown in RPMI Medium 1640 (Gibco) supplemented with 10% fetal bovine serum at 37°C in a humidified 5% CO_2_ atmosphere (Thermo Scientific, USA). Around 2×10^5^ cells were seeded in each well of 24-well plates and cultured overnight. Tissue culture medium was removed and cells were washed with 1640 medium three times before treatment. *V. fluvialis* strains were grown in Brain Heart Infusion broth (BHI, OXOID) supplemented with 0.5% NaCl at 37°C for 18 h in a rotary shaker. The culture supernatant, collected by centrifugation at 8000 rpm for 2 min, was filter-sterilized using 0.22 μm filter units (Millipore, USA). The resultant cell-free culture filtrate was serially diluted and aliquots of each test dilution were added in triplicate to the cell culture plate and incubated for 24 h. Morphological changes and cytotoxic effects were observed after 24 h using an inverted microscope (Nikon ECLIPSE Ti-SR, JAPAN). The toxin titer was expressed as the highest dilution that affected 50% of the cells in a well [21]. The cell pellet was washed and suspended in 1640 medium. Approximately 10^7^ bacterial cells were then added in triplicate to the cell culture plate and incubated for 8 h at 37°C with 5% CO_2_. Cytotoxic activity was detected by Lactate dehydrogenase (LDH) cytotoxicity assays Kit (Promega) according to the instruction of manufacture. 2% Triton X-100 served as positive controls and BHI, RPMI Medium 1640 as negative controls. Cytotoxic activity was calculated according to the following formula: LDH(%)=[OD490_(sample)_ − OD490 _(negative control)_/OD490_(positive control)_ − OD490_(negative control)_] ×100%.

### PFGE

PFGE was performed according to the PulseNet standardized PFGE protocol for *V. cholerae* subtyping [41]. Genomic DNA of *V. fluvialis* strains was prepared in agarose plugs and digested with the restriction enzyme *Not*I, separated in a 1% agarose gel in 0.5×TBE buffer at 14°C using a CHEF-DRIII apparatus (Bio-Rad, Hercules, CA, USA). The pulse time ranged from 2 to 10 s for 13 h, and then from 20 to 25 s for 6 h, both at 6 v/cm. Gels were stained in distilled water containing 1.0 μg ethidium bromide ml^-1^ for 30 min, destained several times and photographed under UV light using the Gel Doc 2000 (Bio-Rad, Hercules, CA, USA). After visualization, the PFGE patterns were analyzed using BioNumerics. Dendrograms were clustered and constructed by using the UPGMA with a tolerance of 1.5%.

### Nucleotide sequence accession number

The nucleotide sequences encoding the enzymes involved in the arginine dihydrolase system and *vfh* gene have been deposited in the GenBank database under accession number KC569550 and KC569551, respectively.

## Results and discussion

### Biological features and biochemical characterizatio

Considering that *V. fluvialis* shares biochemical properties with Aeromonas species and the API 20E system gave ambiguous identity sometimes [26], the identities of *V. fluvialis* were first confirmed by using two sets of species-specific primers which targeted at the conserved transcriptional activation and variable membrane tether regions of the *toxR* gene and 16S-23S rDNA intergenic sequence, respectively [32,33]. Excluding three strains with negative amplifications, 43 strains with both the expected amplicons size were molecular-confirmed as *V. fluvialis* and included in this study. All 44 strains including the reference strain CICC21612 grew as yellow colonies on the TCBS plates and grew well in the presence of 6% salt; however, 36 strains (81.8%) did not grow in LB without NaCl. And 8 strains (18.2%) grew very poorly. Salt tolerance test is very important in distinguishing *V. fluvialis* from the *Aeromonas* species as *Aeromonas* species cannot grow in the presence of 6% NaCl [42]. Variable results were observed in LB with 7% salt: 31 strains (70.5%) grew and 13 strain (29.5%) did not grow. 28 strains (63.6%) were resistant to 10 μg of vibriostatic agent. All the strains were positive in Oxidase and String test, negative in Voges–Proskauer (VP) test, H_2_S production, urease and tryptophane deaminase. They were positive in mannitol, sucrose and arabinose fermentation, negative in inositol, rhamnose and melibiose fermentation. o-nitrophenyl-β-D-galactopyranoside (ONPG) was positive except for strain JS38. Except strains JS50 and Ma-2531 which were arginine dihydrolase-negative, all strains were lysine decarboxylase-negative, ornithine decarboxylase-negative and arginine dihydrolase-positive. ONPG positivity is a generally believed biochemical trait in *V. fluvialis*, but ONPG-negative *V. fluvialis* had been reported [21]. The results of *V. fluvialis* strains in a variety of biological features and API 20E profile numbers are shown in Additional file 1: Table S1.

Arginine dihydrolase -negative *V. fluvialis* strain was not reported in the literature before. The arginine dihydrolase system which converts arginine to ornithine, ammonia and carbon dioxide via citrulline consists of three enzyme reactions catalysed by arginine deiminase, ornithine carbamoyltransferase and carbamate kinase [43,44] and facilitates acid tolerance. Arginine deiminase, i.e. arginine dihydrolase is the first enzyme involved in this system catalysing the chemical reaction: L-arginine + H_2_O ↮ L-citrulline + NH3 [43]. PCR amplification revealed that nucleotide sequences encoding arginine deiminase, ornithine carbamoyltransferase and carbamate kinase are absent in strain Ma-2531. Though strain JS50 displayed negative phenotype of arginine dihydrolase, PCR assay gave the similar-size of amplicon as other strains and sequence analysis revealed no mutation in the enzyme’s coding region, suggesting somehow the expression of the arginine dihydrolase system was affected in JS50. To further exclude the possibility of negative amplification due to sequence variation of the primer annealing regions in strain Ma-2531, additional two primers *arc*-rev and *arc*-ck-up were designed based on the sequence of *arc* operon of *V. fluvialis* strain JS50. Primer pairs *arc*-rev/*arc-*F and *arc*-ck-up/*arc*-R gave the expected size of amplicons in all tested *V. fluvialis* strains except Ma-2531. Sequence analysis of *vfh* gene of Ma-2531 displayed 98% identity to the reference sequence of *V. fluvialis* hemolysin gene in GenBank (AF348455.1), which further confirmed Ma-2531 was an arginine dihydrolase-negative *V. fluvialis*. The corresponding gene sequences encoding the enzymes involved in the arginine dihydrolase system in JS50 and *vfh* gene in Ma-2531 were deposited in the Genbank under accession number KC569550 and KC569551, respectively. Lysine decarboxylase, ornithine decarboxylase, arginine didydrolase, and L-arabinose are often used as the species-specific minimal biochemical tests to identify *V. fluvialis* from *V. cholerae* and nonagglutinating (NAG) vibrios [4]. The appearance of arginine didydrolase-negative *V. fluvialis* increased the complexity of the identification through biochemical tests.

### Identification of virulence genes and phenotypes

Several virulence factors important in pathogenesis have been reported in *V. fluvialis*[15]–[22]. Though the precise role in producing the clinical manifestations remains unclear, these factors may increase the pathogenicity of *V. fluvialis* and contribute to diarrhea. We screened the presence of the virulence genes *vfh*, *hupO*, *vfpA* and *stn* by PCR. All strains were positive for genes *vfh*, *hupO* and *vfpA*, negative for gene *stn* encoding the toxin NAG-ST enterotoxin [37].

**Figure 1:**
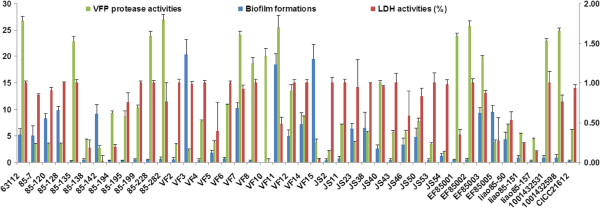
**The virulence phenotypes of *V. fluvialis *including VFP protease production, cytotoxic activity and biofilm formation.**

Azocasein assay was used to determine the product of VFP protease (Figure 1). 17 (39.5%) strains had medium to high expression of VFP with the asocasein unit values ranging from 10.95 to 26.87, the others showed lower VFP productions with the asocasein unit values below 10. Among the above 17 strains, 13 were isolated from stool samples. In contrast to the prevalence of the *vfp* genes in the all tested strains, the corresponding phenotypes were not detected in an equal rate, suggesting the defective expression of VFP in some strains and needs further study. VFP is 70% homologous to precursor proteins of metalloproteases from other human pathogenic vibrios such as *V. cholerae*[45] and *V. vulnificus*[46]. It shows haemagglutinating, permeability-enhancing and haemorrhagic activities in addition to proteolytic activity like *V. vulnificus* protease [47]. Metalloprotease of *V. cholerae*, also called haemagglutinin/protease (Hap), plays an important role in cholera pathogenesis by proteolytically activating cholera toxin A subunit [48] and the El Tor cytolysin/haemolysin [49], hydrolysing physiologically important proteins [50] and promoting mucin gel penetration, detachment and spread of infection along the gastrointestinal tract [51]. Based on the similar biological activities and high homology to metalloproteases of *V. cholerae* and *V. vulnificus*, VFP may also function as an important pathogenic factor in *V. fluvialis*. Strains with higher expression of VFP could be potentially more virulent in the pathogenesis than those with lower expression or no expression, which was consistent with our observation that clinical isolations were predominant in those with medium to high VFP production.

All strains were agglutination-negative to both the chicken and human erythrocytes. On Columbia blood agar, the colonies of all strains were medium to large, mucoid, gray and β-hemolytic, indicating that VFH was routinely produced. Except JS 23 and liao85-157, all strains showed obvious hemolytic zone after 24 h incubation. Longer incubation (more than 72 h) was needed to develop clear hemolytic phenotype for JS23 and liao85-157. The role and biological properties of hemolysin from *V. fluvialis* have been independently studied by two groups [18,20]. In addition to lysing erythrocytes from different animal species, this enterotoxigenic El Tor-like hemolysin was cytotoxic towards CHO cells and induced fluid accumulation in mouse [18]. Pores formed by VFH in erythrocyte membrane seem larger than those formed by other *Vibrio* hemolysins such as *V. cholera*, *V. parahaemolyticus* and *V.vulnificus*[20], which may be somehow related with the bloody diarrhea [2,5,26] occurred in some patients.

Different with *V. cholerae* and *V. parahaemolyticus*, which isolates from clinical sources are predominately toxigenic by containing virulence genes such as *ctxAB* or *tdh* and *trh*, no correlation between the prevalence of virulence genes and isolation source was observed in *V. fluvialis*. Virulence genes *vfh*, *hupO* and *vfpA* were equally detected in patient isolates and seafood isolates. All test *V. fluvialis* were haemolysis-positive irrespective of the isolation sources. Our results indicate the higher risk and potential public health threaten of seafood contaminated by *V. fluvialis.*

### Cytotoxic activity

The supernatants from all the *V. fluvialis* strains were capable of causing cytotoxic to Hep-2 cell. Cell morphological changes including cell rounding and destruction of the monolayer were readily observed after 24 h treatment. The end-point titres showing cytotoxic effects on 50% Hep-2 cells in a well mainly ranged from 2 to 8, only four (VF7, VF2, Ma-2598 and Ma-2531) showed high titres of 16, 32, 16 and 64, respectively. In contrast to the report of Chakraborty *et al.*[21], vacuolating effect was not clearly observed, maybe due to the variation of strains and the difference of tissue cell line used.

Cytotoxocity was also detected by measuring lactate dehydrogenase (LDH) activities of Hep-2 cells after 8 h treatment with *V. fluvialis*. Cytotoxocity was expressed as LDH released into the medium as a percentage of total cellular LDH, obtained by treatment with 2% Triton X-100. LDH activities varied with strains (Figure 1). 33 strains (75%) displayed media to high cytotoxocity with LDH value between 53.12% and 100%, the rest 11 strains (25%) showed low cytotoxocity with LDH value below 48.5%. The average LDH activity evoked by 10 seafood-isolated strains was 68%, which was no significantly different from average value of 76% caused by 34 clinical isolates. It was found that for the above four strains (VF7, VF2, Ma-2598 and Ma-2531) which had the highest end-point titres of supernatants showing cytotoxic effects, LDH values were also high; for the strains with LDH below 50%, the end-point titres of supernatants showing cytotoxic effects were all between 2 and 4.

### Biofilm formation

*V. fluvialis* was found to be the most predominant species among *Vibrio* isolated from both the suburban and urban community effluents in South Africa [52], suggesting its high capacity of survival and persistence in the environment. Amel *et al.* reported a long-term survival (6 years) of *V. fluvialis* in marine sediment [53]. Therefore the ability to form biofilm in *V. fluvialis* was tested. The biofilm formation varied greatly with different strains (Figure 1), 20 (45.5%) strains could form biofilm in vitro, among which three strains VF3, VF11 and VF 15 isolated from stool samples made very thick biofilms. Microbes in biofilm communities are more resistant to environmental stresses and protozoan predation. Strains with the capacity to form higher biofilms could survive better in the infecting host and estuarine system than those forming less biofilms and thus contribute to the pathogenesis.

### Antibiotic susceptibility test

All *V. fluvialis* strains were found to be sensitive to ceftazidime, chloramphenicol, ciprofloxacin, gentamicin, nalidixic acid, trimethoprim and co-trimoxazole (Table 2). Except JS2, all strains were sensitive to tetracycline. And all strains were sensitive to streptomycin except Ma-2531. JS43 is the only strain showed resistant to the third generation of cephalosporins cefotaxime and ceftriaxone with MIC of 4 μg/ml. Additionally, there were six strains showed intermediate to cefotaxime and ceftriaxone with MIC of 2 μg/ml. 45.5%, 38.6%, 25.0% and 38.7% resistance (including intermediate resistance) to ampicillin, azithromycin, sulfamethoxazole and amoxicillin/clavulanic acid were observed with the corresponding MIC ranged from 16 to 64 μg/ml, 4 to 16 μg/ml, more than 1024 μg/ml, and 16/8 to 64/32 μg/ml, respectively. 12 (27.3%) strains were resistant to two and three antibiotics, if strains showing intermediate were included, the rate was up to 34.0%. Strain Ma-2531 isolated from clinical sample in 2010 was resistant to five antibiotics. Antibiotic susceptibility comparison of strains from Fujian revealed that patient isolates showed higher resistant rate (91.7%) and broader resistant spectrum than the seafood-isolates (30%), which suggests patient isolates may bring a more severe medical and public health concern.

**Table 2:** **Antibiotic susceptibility patterns of*V. fluvialis*strains**

**Antibiotic**	**Breakpoints (mg/ml)**	**Resistance**		**Sensitivity**
		**R (%)**	**I (%)**	**S (%)**
Ampicillin	S<=8 I=16 R>=32^a^	12 (27.3)	8 (18.2)	24 (54.5)
Amoxicillin/Clavulanic acid	S<=8/4 I=16/8 R>=32/16^b^	10 (22.7)	7 (15.9)	27 (61.4)
Cefotaxime	S<=1 I=2 R>=4^b^	1 (2.3)	6 (13.6)	37 (84.1)
Ceftriaxone	S<=1 I=2 R>=4^b^	1 (2.3)	6 (13.6)	37 (84.1)
Ceftazidime	S<=4 I=8 R>=16^b^	0 (0)	0 (0)	44 (100)
Chloramphenicol	S<=8 I=16 R>=32^a^	0 (0)	0 (0)	44 (100)
Ciprofloxacin	S<=1 I=2 R>=4^b^	0 (0)	0 (0)	44 (100)
Gentamicin	S<=4 I=8 R>=16^b^	0 (0)	0 (0)	44 (100)
Nalidixic acid	S<=16 R>=32^b^	0 (0)	-	44 (100)
Streptomycin	S<16 R>=16^d^	1 (2.3)	-	43 (97.7)
Sulfamethoxazole	S<=256 R>=512^a^	11 (25.0)	-	33 (75.0)
Trimethoprim	S<=8 R>=16^b^	0 (0)	-	44 (100)
Co-trimoxazole	S<=2/38 R>=4/76^a^	0 (0)	-	44 (100)
Tetracycline	S<=4 I=8 R>=16^a^	1 (2.3)	0 (0)	43 (97.7)
Azithromycin	S<=2 I=4 R>=8^c^	12 (27.3)	5 (11.4)	27 (61.3)

^a^Breakpoints are based on the CLSI standards for *V. cholerae*.

^b^Breakpoints refer to the CLSI criteria for *Enterobacteriaceae*.

^c^Breakpoint is based on the CLSI standards for *C. jejuni.*

^d^Breakpoint refer to the reference [61].

R, resistance; I, intermediate; S, sensitivity.

Compared to the high sensitivity (99.4%) of *V. cholerae* to the ampicillin during the similar time period (1977–1989) [55], *V. fluvialis* exhibited much higher resistant to β- lactams. In regard to sulfamethoxazole resistance, *sul*II gene was detected in 85–142, JS2 and Ma-2531 strains. Resistance to the azithromycin (38.7%, including intermediate resistance) is the unique feature of the *V. fluvialis* tested in this study, strain 63112 isolated in 1963 from a diarrheal patient also showed azithromycin resistance with MIC of 8 μg/ml. Wang *et al.* reported that the earliest azithromycin-resistant *V. cholerae* strains appeared in 1965 in China [55]. Considering the isolation time of the strains and the time-to-market of the second generation macrolides antibiotics, we reasoned that azithromycin-resistant phenotype of *V. fluvialis* in this study was due to the cross-resistance caused by the first generation macrolides antibiotics which had been widely used in the clinical since 1952.

In general, the antibiotic resistant conditions of our *V. fluvialis* strains were not as serious as those reported in the literatures where the SXT element, plasmid and integrons mediated MDRs were quite common in *V. fluvialis*[3,25,56]–[58]. And *V. fluvialis* was reported to be the most abundant strain harboring most of the antibiotic resistance genes and SXT element among the *Vibrio* strains isolated from wasterwater final effluents [59]. The probable reason was the earlier isolation time. The majority of the tested strains were isolated in 1980s. It was reported that integrons appeared in *V. fluvialis* after 1998 [25] and SXT was detected for the first time in O139 *V. cholerae* isolated in 1992 [60]. Consistent to the phenotypes of lacking of SXT specific MDR pattern and sensitivity to the aminoglycosides, PCR screening of the SXT integrase and 3’ conserved sequence (3’CS) and 5’CS of class I integron gave no amplicons (data not shown). From the other side, our results further suggest the rapid increasing and spreading of antibiotic resistance in the *V. fluvialis* in recent 20 years, the appearance of MDR strains will be a severe medical and public health problem due to its epidemic-causing potential. It's worth noting that *int* IV gene specific for the class IV integron [34] was negative in all strains, thus suggesting there are maybe no superintegron in the *V. fluvialis* or sequence variation occurred in the *int* IV gene.

### PFGE

**Figure 2:**
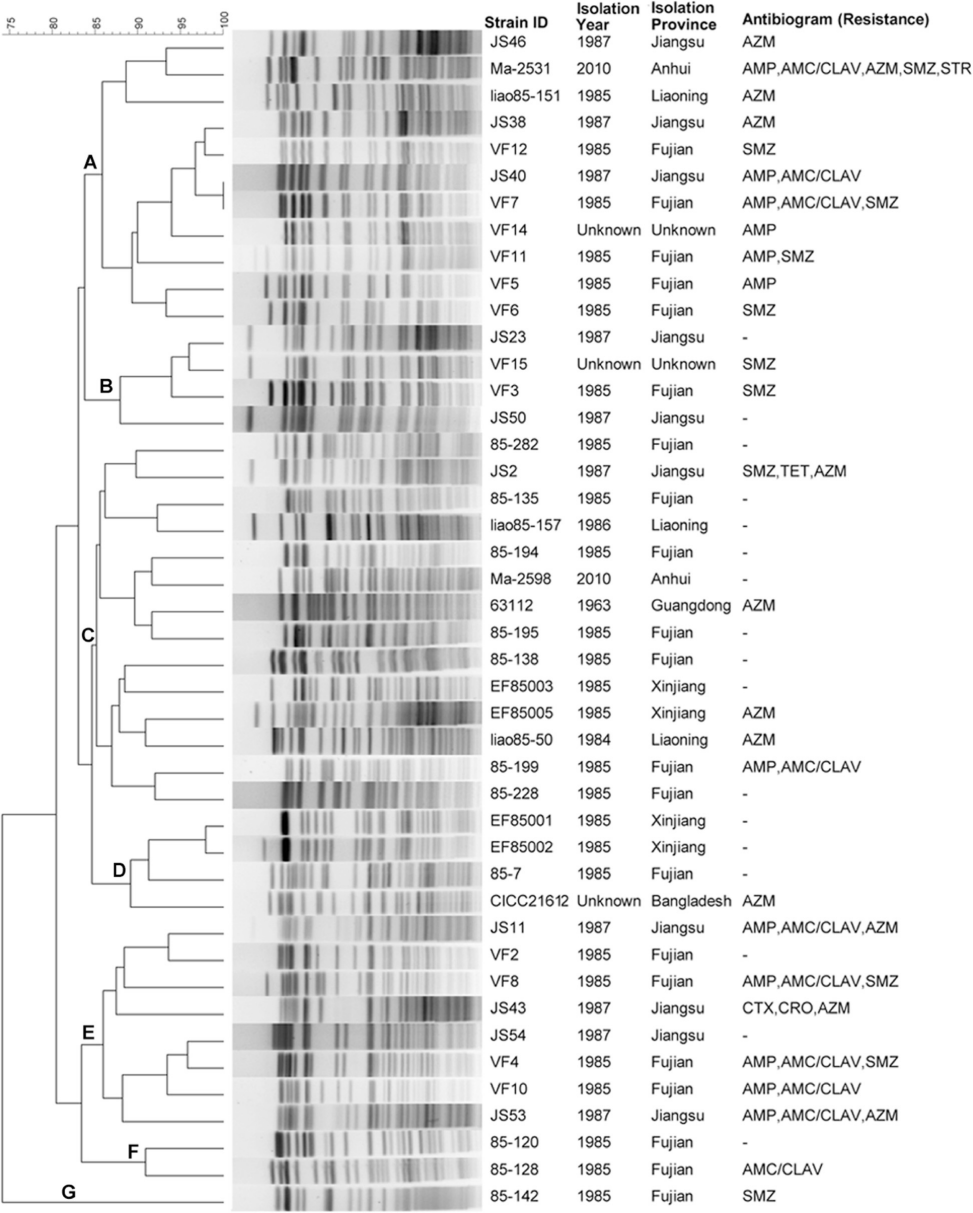
**The results of the PFGE analysis using *No t*I digestion of *V. fluvialis *strains and the MDR patterns.** The dendrogram was produced using the Dice coefficient and the unweighted-pair group method with an arithmetic mean algorithm (UPGMA) with a position tolerance of 1.5%. Abbreviation: AMP, ampicillin; AMC/CLAV, amoxicillin/clavulanic acid; CTX, cefotaxime; CRO, ceftriaxone; STR, streptomycin; SMZ, sulfamethoxazole; TET, tetracycline; AZM, azithromycin.

PFGE was used to analyze the genetic relatedness, molecular-subtyping characteristics of *V. fluvialis* strains, especially to determine whether some strains from the same location during the same year were clonal. PFGE of the *Not*I-digested DNA of 44 strains generated 43 distinct patterns, only two (JS40 and VF7) possessed the same pattern (Figure 2). All strains formed 7 clusters at the 85% similarity breakpoint. The top two clusters contained 14 and 11 strains respectively. The smallest cluster only contained one strain. Five out of 11 strains in cluster A were patient isolates from Fujian in 1985, and 7 out of 14 strains in cluster C were seafood isolates from Fujian. Cluster C exhibited less antibiotic resistance than Cluster A, the most strains in cluster E displayed multiple antibiotic resistance. According to the interpretive criteria proposed by Tenover [61], strain EF85001 and EF85002 were considered to be closely related by showing one band difference which were isolated from same location at same time, strain 85–199 and 85–228, VF5 and VF6, 85–120 and 85–128 were possibly related.

## Conclusions

In this study, we examined the main biological characteristics, virulence phenotypes and their correlation with genetic factors, drug resistance profiles of *V. fluvialis* isolated from patients and environment in China. One strain was found to be negative in arginine dihydrolase system. There was no significant correlation between the prevalence of virulence phenotypes and isolation source. Virulence genes *vfh*, *hupO* and *vfpA* were widely distributed, the ability to produce hemolysin, cytotxin and protease varied with strains. Resistance to β-lactams and Sulfamethoxazole were prevalence. Azithromycin resistance is a unique feature of the *V. fluvialis* tested in this study. PFGE-based comparative molecular analysis of isolates demonstrated great genetic heterogeneity of *V. fluvialis* in China. To our knowledge, this is the first study that specifically evaluated etiological characteristics and molecular relatedness of *V. fluvialis* isolated in China. The obtained information contributed to the understanding of pathogenicity and the epidemiological features of *V. fluvialis* and it’s necessary to enhance surveillance in the future due to the increasing appearance of MDR strains and its epidemic-causing potential.

## Abbreviations

PCR: Polymerase chain reaction; TCBS: Thiosulphate citrate bile salts sucrose agar; VFP: *V. fuvialis* protease; CLSI: Clinical and Laboratory Standards Institute; MICs: Minimum inhibitory concentrations; LDH: Lactate dehydrogenase; PFGE: Pulsed-field gel electrophoresis; ONPG: O-nitrophenyl-β-D-galactopyranoside; VFH: *V. fluvialis* hemolysin.

## Competing interests

The authors declare that they have no competing interests.

## Authors' contributions

PL carried out the molecular genetic studies and phenotypes test, participated in the sequence submission and drafted the manuscript. XC carried out the tissue culture. XD participated in the PFGE analysis. BK participated in the design of the study and helped to draft the manuscript. WL conceived of the study, and participated in its design and coordination and drafted the manuscript. All authors read and approved the final manuscript.

## Authors' information

State Key Laboratory for Infectious Disease Prevention and Control, and National Institute for Communicable Disease Control and Prevention, Chinese Center for Disease Control and Prevention. Beijing 102206, People’s Republic of China.

## Supplementary Material

Additional file 1: Table S1 Information and biological features of *V. fluvialis* strains used in this study. Click here for file
