# Supplementary material for: The virulence phenotypes and molecular epidemiological characteristics of Vibrio fluvialis in China
Source: Gut Pathog. 2013 Mar 22;5:6. doi: 10.1186/1757-4749-5-6 (PMC3636005; doi:10.1186/1757-4749-5-6)
Supplement: Additional file 1: Table S1 — Information and biological features of V. fluvialis strains used in this study. [file 1757-4749-5-6-S1.pdf]

Supplemental materials

Supplemental Table Information and biological features of *V. fluvialis* strains used in this study.

Strain	Province	Year	Source	API 20E (profile number)	Susceptibility to O/129 (10μg)	LB (0% NaCl)	LB (1% NaCl)	LB (6% NaCl)	LB (7% NaCl)	*toxR*	VF-LU	*hupO*	*stn*	*vfh*	*vfpA*	*sul*II	*Int*IV	*arc*
63112	Guangdong	1963	patient	3006126	S	–	+	+	+	+	+	+	–	+	+	–	–	+
85-7	Fujian	1985	patient	3004127	R	–	+	+	+	+	+	+	–	+	+	–	–	+
85-120	Fujian	1985	shrimp	3244126	R	–	+	+	+	+	+	+	–	+	+	–	–	+
85-128	Fujian	1985	shrimp	3244126	R	±	+	+	+	+	+	+	–	+	+	–	–	+
85-135	Fujian	1985	fish	3046526	S	±	+	+	+	+	+	+	–	+	+	–	–	+
85-138	Fujian	1985	fish	3244126	R	–	+	+	+	+	+	+	–	+	+	–	–	+
85-142	Fujian	1985	fish	2044126	R	–	+	+	–	+	+	+	–	+	+	+	–	+
85-194	Fujian	1985	fish	2006126	R	±	+	+	+	+	+	+	–	+	+	–	–	+
85-195	Fujian	1985	fish	3046126	R	±	+	+	+	+	+	+	–	+	+	–	–	+
85-199	Fujian	1985	shrimp	3006127	R	±	+	+	–	+	+	+	–	+	+	–	–	+
85-228	Fujian	1985	shrimp	3006126	S	–	+	+	+	+	+	+	–	+	+	–	–	+
85-282	Fujian	1985	shrimp	3046127	S	–	+	+	+	+	+	+	–	+	+	–	–	+
VF2	Fujian	1985	patient	3004127	S	–	+	+	–	+	+	+	–	+	+	–	–	+
VF3	Fujian	1985	patient	3044126	S	–	+	+	–	+	+	+	–	+	+	–	–	+
VF4	Fujian	1985	patient	3044126	S	–	+	+	+	+	+	+	–	+	+	–	–	+
VF5	Fujian	1985	patient	3044126	R	–	+	+	–	+	+	+	–	+	+	–	–	+
VF6	Fujian	1985	patient	3046126	R	–	+	+	+	+	+	+	–	+	+	–	–	+
VF7	Fujian	1985	patient	3046126	S	–	+	+	+	+	+	+	–	+	+	–	–	+
VF8	Fujian	1985	patient	3206127	R	–	+	+	+	+	+	+	–	+	+	–	–	+

VF10	Fujian	1985	patient	3006127	R	–	+	+	+	+	+	+	–	+	+	–	–	+
VF11	Fujian	1985	patient	3046127	R	–	+	+	+	+	+	+	–	+	+	–	–	+
VF12	Fujian	1985	patient	3042126	S	–	+	+	–	+	+	+	–	+	+	–	–	+
VF14	unknown	unknown	patient	3246126	R	–	+	+	–	+	+	+	–	+	+	–	–	+
VF15	unknown	unknown	patient	3044127	S	–	+	+	–	+	+	+	–	+	+	–	–	+
JS2	Jiangsu	1987	patient	3244126	R	–	+	+	+	+	+	+	–	+	+	+	–	+
JS11	Jiangsu	1987	patient	3206126	R	–	+	+	+	+	+	+	–	+	+	–	–	+
JS23	Jiangsu	1987	patient	3004126	R	–	+	+	+	+	+	+	–	+	+	–	–	+
JS38	Jiangsu	1987	patient	2006126	R	–	+	+	+	+	+	+	–	+	+	–	–	+
JS40	Jiangsu	1987	patient	3006126	R	–	+	+	–	+	+	+	–	+	+	–	–	+
JS43	Jiangsu	1987	patient	3004126	R	–	+	+	+	+	+	+	–	+	+	–	–	+
JS46	Jiangsu	1987	patient	3006126	R	–	+	+	–	+	+	+	–	+	+	–	–	+
JS50	Jiangsu	1987	patient	1044127	S	–	+	+	–	+	+	+	–	+	+	–	–	+
JS53	Jiangsu	1987	patient	3044126	R	–	+	+	+	+	+	+	–	+	+	–	–	+
JS54	Jiangsu	1987	patient	3044127	R	–	+	+	—	+	+	+	–	+	+	–	–	+
EF85001	Xinjiang	1985	patient	3206127	S	–	+	+	+	+	+	+	–	+	+	–	–	+
EF85002	Xinjiang	1985	patient	3046126	S	–	+	+	+	+	+	+	–	+	+	–	–	+
EF85003	Xinjiang	1985	patient	3206127	R	–	+	+	+	+	+	+	–	+	+	–	–	+
EF85005	Xinjiang	1985	patient	3044127	S	–	+	+	–	+	+	+	–	+	+	–	–	+
liao85-50	Liaoning	1984	patient	3006126	R	–	+	+	+	+	+	+	–	+	+	–	–	+
liao85-151	Liaoning	1984	patient	3004126	S	–	+	+	+	+	+	+	–	+	+	–	–	+
liao85-157	Liaoning	1984	patient	3004126	S	–	+	+	+	+	+	+	–	+	+	–	–	+
Ma-2531	Anhui	2010	patient	1006126	R	±	+	+	+	+	+	+	–	+	+	+	–	–
Ma-2598	Anhui	2010	patient	3206126	R	±	+	+	+	+	+	+	–	+	+	–	–	+
CICC21612	Bangladesh	unknown	patient	3000127	S	±	+	+	+	+	+	+	–	+	+	–	–	+

+, Positive; —, negative; ±, very poor positive; R, resistance; S, sensitivity
